# Effectiveness of transanal irrigation in low anterior resection syndrome

**DOI:** 10.1111/codi.70193

**Published:** 2025-08-25

**Authors:** Shreya Jauhari, Digby Hopkinson‐Woolley, Karen Curran, Rebecca Doyle, Helen Boffin, Kim Gorissen, Sandeep Singh

**Affiliations:** ^1^ Oxford University Hospitals Trust Oxford UK

**Keywords:** bowel dysfunction, functional outcomes, LARS score, low anterior resection syndrome (LARS), pelvic floor rehabilitation, rectal cancer, transanal irrigation (TAI)

## Abstract

**Background:**

Low anterior resection syndrome (LARS) is a frequent issue leading to bowel dysfunction after anterior resection surgery. NICE guidelines state that there is limited research around the management of LARS. Transanal irrigation (TAI) is a suggested treatment by guidelines; however, there is limited research surrounding the effectiveness of this treatment.

**Aim:**

The aim of this prospective study was to evaluate the effectiveness of TAI in reducing LARS score post anterior resection surgery.

**Methods:**

Patients who were referred to the pelvic floor nurse specialist team between 2019 and 2023 with bowel dysfunction post anterior resection surgery were evaluated. Bowel dysfunction was assessed using the LARS scoring system during the first assessment and on discharge. These patients were offered TAI and trained to perform TAI. Patients with missing LARS scores and those who were not using TAI were excluded from the study.

**Results:**

Of the total 37 patients who were referred and using TAI, 16 were excluded. In total, 21 patients were included (12 male, 9 female). At baseline, 33% of patients were recorded to have minor LARS, and 66% with major LARS. At discharge, there was a significant improvement in LARS score (80% of patients reported no LARS, 10% minor LARS and 10% major LARS). Overall, the LARS score at discharge was significantly lower among patients who underwent irrigation (mean 34.57 vs. 12.48, *p* = 0.0000000038).

**Conclusion:**

Our study shows the importance of TAI in the management of bowel dysfunction post anterior resection for rectal cancer, with more than two‐third of patients' symptom improvement.

**What does this paper add to the literature?**

Due to limited research surrounding transanal irrigation (TAI) in low anterior resection syndrome (LARS) management, this study aimed to evaluate the effectiveness of TAI in managing LARS post anterior resection for rectal cancer. Overall, our study shows the importance of TAI, with more than 2/3rd of patients' symptom improvement.

As per the GLOBOCAN 2020 study, rectal cancer is stated to be the third most common cancer in the world [1]. Around 52% of patients with rectal cancer will undergo major resectional surgery [2]. The majority of patients who undergo surgery report having ongoing bowel dysfunction post the surgery [3, 4, 5, 6]. These clusters of symptoms post anterior resection have been termed as low anterior resection syndrome (LARS). LARS consists of a plethora of symptoms associated with bowel dysfunction, with a recent international consensus defining LARS as having at least one of eight symptoms resulting in at least one of eight consequences after anterior resection [7].

It is reported that the prevalence of LARS is around 60%–90% after low anterior resection syndrome, with studies suggesting it to have a significant impact on quality of life [3, 4, 5, 6, 8]. Long‐term studies have shown the presence of LARS to persist in a large proportion of patients up to 15 years after anterior resection [8, 9, 10]. The pathophysiology surrounding LARS is still uncertain; however, there is developing evidence suggesting that LARS has a multifactorial aetiology comprising several components, leading to a complex mixed pathophysiological model [6].

Despite its large prevalence, guidelines and recommendations are currently based on limited evidence‐based medicine [11]. Traditional management of LARS has included options such as pelvic floor exercises and dietary modifications; however, there has been increasing interest in further exploring the effectiveness of transanal irrigation (TAI) as an alternative management option [12]. Previously, TAI was researched for functional bowel disorders and neurogenic bowel conditions, until being considered for patients with LARS [13].

TAI involves using an irrigation system to introduce water to wash out the rectum at timed intervals to offer symptomatic relief, with the aim to reduce LARS symptoms and improve the patient's quality of life as a result of regular bowel emptying [14]. It has been insinuated that regular TAI practices may improve colonic motility due to the high pressure water systems present in TAI stimulating motility [15]. TAI in the treatment of LARS has shown positive results in small‐scale studies [15, 16, 17]. Due to the limited research surrounding the effectiveness of TAI in the management of LARS, the aim of this prospective study is to evaluate the effectiveness of TAI in managing LARS in patients post low anterior resection.

## METHODOLOGY

### Study population and design

This prospective study evaluated patients in the care of a single centre. It includes patients who were referred to the pelvic floor nurse specialist team between 2019 and 2023 with bowel dysfunction post anterior resection surgery. Of these patients, only patients who made a decision to carry out transanal irrigation were included. Of these patients, any who did not have stoma reversal, were not discharged, or who had missing data were further excluded, as seen in Flow Chart 1.

**FLOW CHART 1:**
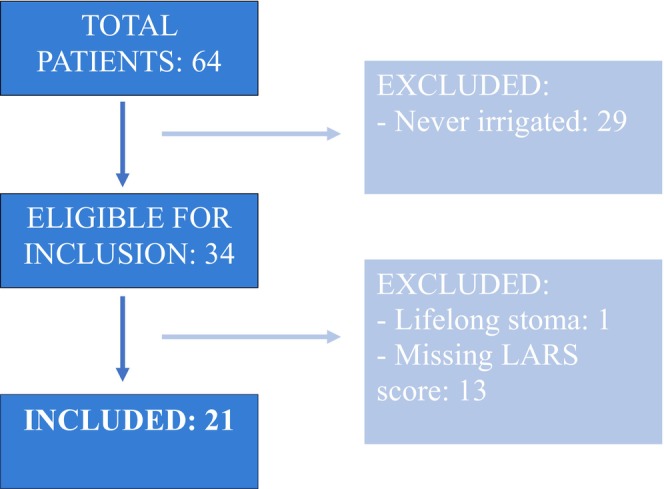
Describing the process of inclusion and exclusion of patients for the study. LARS, low anterior resection syndrome.

### Low anterior resection syndrome questionnaire

Bowel dysfunction was assessed using the LARS scoring system during the first assessment and on discharge. The validated LARS score questionnaire was used to evaluate the LARS score for included patients [18]. There has been a correlation between no LARS and major LARS with an impact on quality of life [19]. This consisted of five questions which assess flatus, accidental leakage, bowel frequency, clustering and urgency. Each of the five questions has three possible answers, each scoring a different number of points. Totalling the score from the answers will allow a patient to be categorised into symptoms of no LARS (0–20 points), minor LARS (21–29 points), or major LARS (30–42 points) [18]. This questionnaire was used at the beginning of the pelvic floor nurse specialist evaluation of LARS prior to starting transanal irrigation and then used at discharge to assess patient symptoms pre and post transanal irrigation.

### Transanal irrigation

TAI was offered to patients during consultation with pelvic floor nurse specialists after being identified as having high LARS scores post anterior resection. During the consultation, TAI was offered to patients and they were trained in how to carry out TAI as per the manufacturer's guidelines until they could independently carry out irrigation without difficulty at home. All patients received standardised training in TAI using the Peristeen®, Peristeen® Plus Cone or Qufora® systems, with 600–800 mL of lukewarm tap water administered once daily. Device selection and irrigation volume were tailored to patient preference and tolerance. Follow‐up sessions were organised at intervals with pelvic floor nurse specialists to support patient progress with the irrigation systems. Finally, the LARS score was calculated at discharge.

Conservative management advice was delivered by specialist pelvic floor nurses during clinic appointments, which included guidance on pelvic floor exercises, toileting techniques and establishing regular bowel routines. Patients also received basic education on hydration, fibre intake and medication review to support bowel function. In accordance with standard protocol, patients were advised to take low‐dose loperamide 15 min prior to meals to support continence daily alongside TAI. No additional adjunctive therapies, such as biofeedback, were employed.

Patients in this study used either Peristeen®, Peristeen® Plus Cone or Qufora® irrigation systems. The Peristeen® and Qufora® irrigation systems consist of a control unit which is also attached to a pump that can regulate air entry for inflation of a rectal balloon and water entry for irrigation of the colon. The Peristeen® Plus Cone system involves a similar system; however, it comes with a cone catheter which does not require inflation of a rectal balloon.

### Statistical analysis

Data were prospectively recorded in a dedicated database. Statistical analysis was performed in Excel. The difference between pre‐ and post‐TAI data were analysed by a paired two‐sample *t*‐test. A difference was considered statistically significant for *p*‐values <0.05.

### Ethical approval

Appropriate ethical approval was sought and obtained for this study.

## RESULTS

Of the total 35 patients who were referred to pelvic floor nurses and using TAI, 14 were excluded. In total, 21 patients were included (12 male, 9 female). Patients had a mean age of 58.95 years. No patients included in the study had undergone chemoradiotherapy.

Patients were further classified into whether they were defunctioned versus not defunctioned (Table 1). Defunctioned patients were further subclassified on whether they had stoma closure <6 months or ≥6 months from anterior resection. The mean time to closure for defunctioned patients to stoma closure in this cohort was 7.24 months. There was no significant difference between the time from anastomosis or closure to the first irrigation appointment with pelvic floor nurses. However, the mean months for a defunctioned patients who had a stoma closure ≥6 months from anterior resection to receive a first irrigation appointment was shorter (9.75 vs. 23.75 and 25.33).

**TABLE 1:** Comparison of patients not defunctioned versus defunctioned.

	Not defunctioned	Defunctioned; stoma closure <6 months	Defunctioned; stoma closure ≥6 months
Number	4	9	8
Age; [mean (SD)]	66.25 (12.01)	57.24 (16.70)	57.56 (13.50)
Time to closure; [mean (SD)]	–	7.24 (4.25)	
Months between anastomosis/closure to first irrigation appointment; [mean (SD)]	25.75 (33.00)	25.33 (36.28)	9.75 (13.14)

Further evaluation of risk factors of developing LARS was explored in the study. Initial LARS scores of patients were compared with gender and age separately. Between both groups, no statistical significance was found. In addition, the study compares defunctioned versus not defunctioned patients' baseline LARS score, which also showed no statistical significance. Defunctioned patients were further classified into stoma closure <6 months or ≥6 months from anterior resection, which again showed no statistical significance.

**GRAPH 1:**
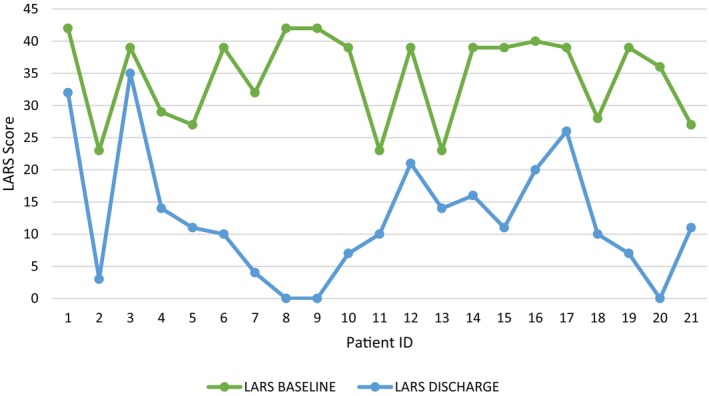
Line graph depicting LARS at baseline versus LARS at discharge pre‐ and post‐TAI. LARS, low anterior resection syndrome; TAI, transanal irrigation.

At baseline, it was found that 33% of patients were recorded to have minor LARS and 66% with major LARS (Bar Chart 1). At discharge, there was a significant improvement in LARS score (80% of patients reported no LARS, 10% minor LARS and 10% major LARS). This is represented in Graph 1. The mean LARS score was lower at study termination, with an average LARS score reduction of −66%. The LARS score at discharge was significantly lower among patients who underwent irrigation (mean 34.57 vs. 12.48, *p* = 0.0000000038). In addition, 17 of the original 21 found ongoing benefit from TAI beyond this and were reported to still be irrigating beyond discharge.

**BAR CHART 1:**
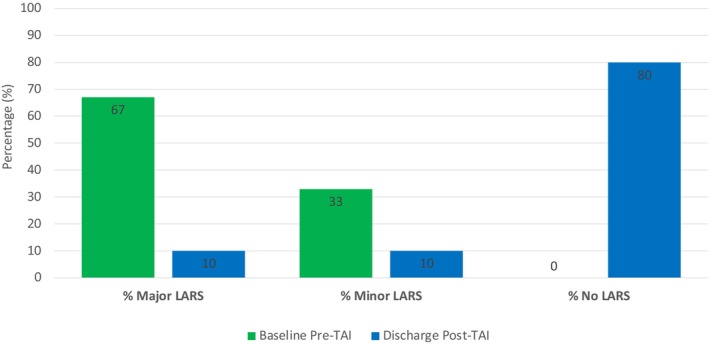
Percentage LARS classification.

## DISCUSSION AND CONCLUSIONS

The effectiveness of TAI in rectal cancer patients post anterior resection is an area with limited research [12]. Current research shows positive effects of TAI in reducing LARS scores post anterior resection [12, 15, 16, 17]. This study aims to build on current literature and explore the effects of TAI in patients with established bowel dysfunction post anterior resection surgery. This study echoed current research, with patients reducing LARS scores by two‐thirds and 80% showing no LARS after the use of TAI.

The POLARS study explores risk factors associated with LARS, which our study attempted to look into further [20]. These risk factors included age and gender to potentially affect the incidence of LARS. Our study found age and gender were not associated with LARS, which is in agreement with Ye et al. [21]. Ye et al. carried out a meta‐analysis including a total of 5102 patients looking at risk factors for the development of LARS [21]. Other factors in Ye et al.'s study, including tumour height and low anastomotic height, could not be evaluated due to limited data in this study.

Further analysis was carried out on patients who were defunctioned versus not defunctioned. This study did not find a correlation with patients being defunctioned versus not defunctioned as being associated with LARS score. This has been echoed in the current literature [22]. Patients defunctioned early versus late also showed no difference in this study, which is in line with some studies [22, 23, 24]. For example, the study by Keane et al. randomised more than 100 defunctioned patients after low anterior resection surgery to early and late and found no reduction in LARS [24]. It is important to note classification of ‘early’ and ‘late’ closure appears to vary in different papers. However, incidence of LARS in defunctioned patients and a prolonged time to anastomosis closure was found to reinforce a negative effect on bowel function in a 2021 meta‐analysis by Vogel et al. [25] The POLARS study also reports fashioning a defunctioning stoma to impair bowel function [20]. Due to our limited sample size further multicentre studies would be needed to further explore these results.

This study also found a correlation between defunctioned patients who had closure ≥6 months from anterior resection having a sooner appointment with the pelvic floor nurse specialists. This raises the possibility of this subgroup developing LARS earlier post anterior resection than others. Time to development of LARS is not overtly explored in the current literature. Further exploration into the timing of symptom development may allow earlier intervention and support for these patient subgroups.

Interestingly, it was found that a large total of 29 individuals chose not to carry out TAI after initial consultations with pelvic nurse specialists. This is a theme also seen in Rosen et al.'s 2020 paper [26]. This paper finds at 12 months follow‐up, when patients are asked to choose between TAI and supportive management, the majority opted towards supportive treatment. This is despite TAI showing a greater reduction in bowel movements. Rosen et al.'s paper highlights that although TAI is promising, a considerable number of patients still stopped TAI within 12 months. They identified individual patient complaints surrounding pain and excessive time consumption when TAI was used regularly [26]. However, despite this, this study found of the 21 patients who decided to irrigate, 17 patients were found to still use TAI beyond discharge. Although not in the remit of this study, future qualitative assessment of the patient experience, stigma and views surrounding TAI is needed to evaluate irrigation compliance and to continue to make TAI more patient‐focused. Recent work by Rethy et al. highlights long‐term benefits of TAI, including sustained symptom relief and reduced reliance on loperamide. Key themes were identified around patient adjustment, routine integration and perceived improvements in autonomy, underscoring the value of incorporating patient experience into future research [27].

Strengths of our study include the use of standardised training via the pelvic floor nurse specialists for all patients in the study, which aimed to increase homogeneity. In addition, the option of an irrigation system and the ability of patients to personalise their treatment were able to offer a real‐world ability to evaluate the effectiveness of TAI. Although limited by its small sample size and non‐randomised design, this study offers preliminary, real‐world evidence of the benefits of TAI in managing LARS and can serve as a foundation for larger, controlled trials.

Limitations of this study include that this was a small, single centre study and it was limited by missing data. Although results are statistically significant, they should be interpreted with caution. Larger, multicentre trials are necessary to confirm these findings. In addition, the validated LARS score was used to evaluate functional bowel dysfunction using a score based on Emmertsen et al.'s 2012 study [18]. This has been further emulated to show correlation between LARS and quality of life in other studies since then [8, 19]. However, a pilot study found 24% of major LARS patients did not experience bowel dysfunction, suggesting LARS score may underestimate the impact of bowel dysfunction and therefore not accurately assess the impact of symptoms on an individual patient's quality of life [28, 29]. A recent 2023 study compared the LARS score with the patient‐reported bowel‐related quality of life (BQOL) impairment using Short Form‐36 to find a high amount of discordance between them, conferring that patient‐reported BQOL may be a better measure for patients than the LARS score [30]. Therefore, using a single marker of assessing severity of LARS may have therefore limited the true impact of TAI in this study. Future studies should consider complementing the LARS score with additional patient reported outcome measures to capture broader aspects of bowel function and quality of life.

Overall, it is evident that the battle with rectal cancer for patients does not cease after LAR surgery. The field of more effective symptomatic management of LARS is an exciting field with a lot of debate. Although TAI has shown to be positive in this study, certain questions still persist to evaluate the effectiveness of TAI, such as (1) volume of irrigation, (2) intervals, (3) irrigation device, (4) qualitative assessment of the patient experience with ongoing follow up and (5) if TAI could be reduced or stopped over time.

In conclusion, TAI is a promising treatment option for patients with LARS post anterior resection. Our study shows the importance of TAI in the management of bowel dysfunction post anterior resection for rectal cancer, with more than two‐third of patient symptom improvement. However, further research is required to further refine and optimise its role in the treatment of LARS and in improving quality of life and outcomes for patients with LARS. In particular, further focus on the patient experience may be able to give us further insight into the effectiveness for this cohort.

## AUTHOR CONTRIBUTIONS

**Shreya Jauhari:** Conceptualization; writing – original draft; writing – review and editing; methodology; formal analysis; project administration; data curation; visualization; investigation; software; resources. **Digby Hopkinson‐Woolley:** Methodology; data curation; investigation; validation. **Karen Curran:** Methodology; investigation; visualization; validation. **Rebecca Doyle:** Methodology; investigation; visualization; validation. **Helen Boffin:** Methodology; investigation; visualization; validation. **Kim Gorissen:** Investigation; methodology; visualization; supervision. **Sandeep Singh:** Supervision; conceptualization; data curation; resources; project administration; formal analysis; software; writing – review and editing; visualization; methodology; investigation; validation; funding acquisition.

## FUNDING INFORMATION

No funding or sponsorship has been received for this study.

## CONFLICT OF INTEREST STATEMENT

The authors declare no conflicts of interest.

## ETHICS APPROVAL STATEMENT

After appropriate ethical approval, our study was registered as service evaluation (nr. 5201).

## PATIENT CONSENT STATEMENT

Individual patient consent to participate in the study was recorded.

## Data Availability

The data that support the findings of this study are available from the corresponding author upon reasonable request.
